# Investigation of Fibers Reinforced Engineered Cementitious Composites Properties Using Quartz Powder

**DOI:** 10.3390/ma13112428

**Published:** 2020-05-26

**Authors:** M. S. Liew, Muhammad Aswin, Kamaluddeen Usman Danyaro, Bashar S. Mohammed, A. M. Al-Yacouby

**Affiliations:** 1 Department of Civil and Environmental Engineering, Universiti Teknologi PETRONAS, Bandar Seri Iskandar 32610, Malaysia; shahir_liew@utp.edu.my (M.S.L.); bashar.mohammed@utp.edu.my (B.S.M.); ahmad.alyacouby@utp.edu.my (A.M.A.-Y.); 2 Departemen Teknik Sipil, Universitas Sumatera Utara, Medan 20155, Indonesia; 18Aswin69@gmail.com; 3 Offshore Engineering Centre, Universiti Teknologi PETRONAS, Bandar Seri Iskandar 32610, Malaysia

**Keywords:** quartz powder, fresh properties, engineered cementitious composites, fibers reinforced engineered cementitious composites, PVA fiber, steel fiber

## Abstract

In relation to the use of retrofit materials on damaged constructions, application on earthquake-resistant buildings, and for the strengthening and rehabilitation on weakened regions, there is a need for a more superior material than concrete. Application sites include beam-column joints, corbels, link-slabs, deep beams, support regions and dapped-end areas. Fiber reinforced engineered cementitious composites (FR-ECC) can address this issue, because FR-ECC is one of the composite materials that has high strength, ductility and durability. In order to develop FR-ECC, this study was done to investigate the effect of adding quartz powder on the compressive strength capacity and properties of FR-ECC through the use of polyvinyl alcohol (PVA) and steel fibers. The volume fraction of fiber was set to 0%–2%. To support the friendly environment, FR-ECC uses by-product materials such as fly ash and silica fume, with a cement content less than 600 kg/m^3^. In terms of the experimental investigation on FR-ECC, this work conducted the fresh property tests showing that PVA fibers have quite an influence on ECC workability, due to their hydrophilic behavior. By adjusting the superplasticizer (SP) content, the consistency and high workability of the ECC mixes have been achieved and maintained. The test results indicated that the PVA and steel fibers-based ECC mixes can be classified as self-compacting composites and high early compressive strength composites. Significantly, addition of quartz powder into the ECC mixes increased the compressive strength ratio of the ECC samples up to 1.0747. Furthermore, the steel fiber-based ECC samples exhibited greater compressive strength than the PVA fibers-based ECC samples with the strength ratio of 1.1760. Due to effect of the pozzolanic reaction, the fibers dispersion and orientation in the fresh ECC mixes, so that the cementitious matrices provided the high strength on the FR-ECC samples. During the compression loading, the bulging effect always occurred before the failures of the fibers-based ECC samples. No spalling occurred at the time of rupture and the collapse occurred slowly. Thus, FR-ECC has provided unique characteristics, which will reduce the high cost of maintenance.

## 1. Introduction

Basically, reactive powder concrete (RPC) is almost similar to engineered cementitious composites (ECC). The basic concept of RPC was first introduced in France in the early 1990s by the researchers of Bouygues Laboratory [1]. RPC was developed with a concept of ultra-high strength concrete. It was created without using the coarse aggregates. RPC contains the high-volume cement, supplementary cement materials (such as silica fume), micro-silica sand, very low water to binder ratio, superplasticizer (SP) and addition of steel fibers. Removal of the coarse aggregates can make RPC able to increase homogeneity between the matrix and aggregates.

By using the available materials in Egypt, Abdelalim et al. [2] reported that RPC can be produced with high mechanical properties, in which they achieved a compressive strength up to 160 MPa and a flexural strength up to 46 MPa. The water-cement ratio of mixes was ranged from 0.17 to 0.19. Silica fume and quartz powder were imperative materials to be utilized. The contents of silica fume and quartz powder were in the ranges of 15%–25% and 30%–40% of the cement weight respectively. However, Khadiranaikar and Muranal [3] have developed the RPC mixes with the certain constituent materials. The materials include cement of 900 kg/m^3^ that was kept constant for all the mixes. The water-binder ratio was varied from 0.16 to 0.24 with SP of 1 to 4%. Silica fume of 15% to 25% and quartz powder of 20% with the cement weight added into the mixes. RPC achieved the maximum compressive strength of 146 MPa, when the silica fume content was 15%. Nonetheless, the addition of quartz powder has resulted in increments of compressive strength up to 20%. Furthermore, Fehling [4] has stated that the range of compressive strength of ultra-high performance concrete (UHPC) was 150 to 220 MPa.

ECC was invented first by Li in the early 1990s at Michigan University, USA. Li et al. [5] and Li [6] developed ECC based on the micromechanics principle, fiber crack bridging behavior, multiple cracks and pseudo-strain hardening. In developing ECC, the fundamental approach is commonly aimed to generate the mutual mechanical interactions between the fiber, matrix, and fiber-matrix interface, as well as to improve ductility and the tensile strength. Their fiber content would be 2% or less by volume fraction [6,7]. High volume fly ash and micro-silica sand were always utilized to support the unique behavior of ECC. Adjusted SP was used to control workability of ECC [8]. Due to the adverse effect on the unique ductile behavior of composites, coarse aggregates were not utilized in the ECC mixtures. In general, the ECC composition consists of a water-cement ratio and sand-cement ratio of 0.5 or lower, where the cement content is less than 600 kg/m^3^. Aside from that, the typical fibers used are a type of polyvinyl alcohol (PVA), but other fibers can also be applied, such as high strength polyethylene (PE) fiber or polypropylene (PP) fiber [9,10]. The main mechanical properties of ECC are exhibited in Table 1 [7].

For different types of infrastructure, cementitious materials are the most broadly used as development materials. However, the characteristic of the brittleness behavior of the cementitious materials was always indicated by the ratio of the low strain and low elasticity to the failure load capacity [11]. Further, ECC was able to develop the numerous cracks evenly over the specimen’s length, which means the opening of each crack can be maintained to less than 100 μm, and more than 2% of the ultimate tensile strain can be reached. The threshold of ECC’s tensile strength had ranged from 4 to 6 MPa, with the compressive strength between 30 and 80 MPa. These depend on the mix design with compressive strain capability of 0.4% to 0.65% [12].

By reviewing the fatigue loading, ECC can provide higher fatigue life compared to the conventional concrete. Many researches have proven that the high performance of ECC can resist better fatigue loading [13]. In addition, ECC has another benefit compared to the conventional concrete, where the narrow crack width feature is less than 100 μm. These crack widths can survive during the ECC stress-hardening phase that make it extremely durable under various adverse conditions [14]. Investigation of ECC that placed in the dapped-end region of beams showed that ECC can improve shear behavior and strength capacity of dapped-end beams [15]. ECC is good enough to be used for RC buildings located in the earthquake zones [12]. Thus, ECC is a compact composite that has a low porosity, which can prevent penetration of liquid or solution that causes rust on steel reinforcements.

In general, the volume fraction of ECC’s fibers is maintained at not more than 2% [16]. The use of polyethylene fibers generates obstacles in fiber dispersion during the mixing. This condition can cause decrement of compressive strength when the fiber contents increased. Likewise, Pan et al. [17] investigates the possibility of utilizing un-oiled and hybrid fibers in ECC. They proved that ECC with un-oiled polyvinyl alcohol (PVA) fibers have a greater ability in the tensile strain. Other fibers such as polyethylene (PE), polypropylene (PP) fibers and the steel fibers are non-polymeric fibers used in ECC. The ECC characteristic depends on the type, geometry and volume of the material substrates in the ECC mixtures [18]. ECC based PVA fibers can develop the powerful chemical bond with the cement matrix in ECC. This is because of their better hydrophilic behavior compared to other fibers. Accordingly, a lot of scientists utilized PVA fibers in the ECC mixtures [19,20]. Due to the existence of hydroxyl groups in the PVA chemical composition, it can develop the chemical bonding between PVA fibers and ECC matrices. This bonding is very strong, making the PVA fibers always rupture first, rather than making the fibers pull-out during the loading [19]. ECC was frequently made up by various contents or sizes of PVA and steel fibers that address the micro and macro cracks along with providing enhanced dynamic characteristics [21]. Mohammed et al. [16] invented the self-compacting ECC modified by nano silica and demonstrated the enhanced hardening characteristics, such as compressive strength, elasticity modulus, and flexural strength without deterioration of ductility or strain-hardening characteristics. In essence, the high-performance ECC could be selected as the construction materials in multiple infrastructures to complement ordinary concrete.

Each year, the average cement production worldwide is more than 4 billion tons, producing around 8% of CO_2_ emissions [22]. This is the serious issue on environment. In most cases, the cement content of RPC is quite high, which generally larger than 700 kg/m^3^. Therefore, this study has been focused primarily to investigate ECC with the cement content less than 600 kg/m^3^. Aside from that, this research also investigates effect of the quartz powder addition on the compressive strength capacity and properties of the fiber reinforced engineered cementitious composites (FR-ECC) with the fiber volume fraction of 0%–2% based on the PVA and steel fibers. Significantly, FR-ECC can be used for the earthquake-resistant buildings application, retrofit materials as well as strengthening and rehabilitation on the disturbed regions such as beam-column joint, corbels, link-slab, deep beam, support region, dapped-end area, etc. FR-ECC is superior to the normal concrete, thus, FR-ECC needs to be further investigated and developed.

## 2. Materials and Methods

### 2.1. Materials

The materials utilized in this work consist of the ordinary Portland cement (OPC), fly ash (FA), densified silica fume (SF), quartz powder (QP), washed river sand (WRS), water, superplasiticizer (SP), polyvinyl alcohol (PVA) and steel fibers.

#### 2.1.1. Cement and Cementitious Materials

The OPC Type-1 used is a product of Tasek Sdn. Bhd., Kuala Lumpur, Malaysia. Fly ash (Class-F with high content of CaO) as the source of aluminosilicate materials was acquired from the Manjung Power Plant, Manjun, Malaysia. Meanwhile, the silica fume and quartz powder used in this research was by-product materials of Malaysia manufacturers, Kanapathy Supplier, Kuala Lumpur, Malaysia. The chemical contents of cement, fly ash, silica fume and quartz powder used were detected by X-ray fluorescence (XRF, Bruker—S8 Tiger model, Billerica, MA, USA), as presented in Table 2.

#### 2.1.2. Fine Aggregate

The local river sand from Sungai Perak Malaysia was used as filler in these ECC mixtures. The local fine aggregate called washed river sand (WRS) was utilized deliberately, so that Malaysian users could get it easily. The particle sizes ranged from 0.3 to 1.2 mm (or 300 to 1200 μm) with a fineness modulus of 2.72.

#### 2.1.3. Fibers

The PVA fibers of the type RFS400 were used with diameter of 0.2 mm, length of 18 mm, specific gravity of 1.3 and tensile strength of 1000 MPa. Moreover, the physical properties of the micro-steel fibers supplied by Dura Technology Sdn. Bhd. (Chemor, Malaysia) include a 0.2 mm diameter, 20 mm length, 2300 MPa tensile strength and specific gravity of 7.86. The steel fibers type used is the hooked ends. Physical properties of both fibers are shown in Table 3.

#### 2.1.4. Superplasiticizer (SP)

A polycarboxylate-based high range water reducer was produced by Sika Kimia Sdn. Bhd. with a familiar product name of Sika^®^ ViscoCrete^®^-2044 (VC-2044, Sika Kimia Sdn. Bhd, Negeri Sembilan, Malaysia). This superplasticizer (SP) was especially used in the FR-ECC mixtures because FR-ECC uses a low water-binder ratio. Characteristic of SP is presented in Table 4.

### 2.2. Experimental Work Plan

This study was advanced from the previous research done by Mohammed et al. [23]. The works exhibited that ECC-1 has been created based on a criterion of the high-volume fly ash, whilst ECC-2 was developed from ECC-1 using moderate-volume fly ash modified by silica fume. Both ECCs were reinforced by PVA fibers. In the meantime, ECCs in this research were derived from ECC-2 which involve the constant addition of quartz powder of 2.5% and varies fraction of PVA or steel fibers. Further, the experimental program is divided into four stages. In the first stage, the properties of ECC containing the PVA fibers with the zero-quartz powder are studied. In the second stage, it was continued to evaluate properties of the PVA fibers reinforced ECC by adding the quartz powder. The mixes proportions of the first and second stages are exhibited in Table 5 and Table 6 respectively. Furthermore, the third stage was characterized by ECC that was produced using steel fibers (instead of PVA) and without quartz powder. The mix proportion can be seen in Table 7. For the last stage, the properties of ECC made with steel fibers with added quartz powder were investigated. The mixes of this stage have been shown in Table 8. The fibers content added to each mix were 0%; 0.5%; 1%; 1.5% and 2% correspondingly for PVA or steel fibers. In order to maintain the high workability of ECC, the amount of SP had been adjusted for each mix in this study.

### 2.3. Mixing Procedure and Specimen Preparation

**Figure 1:**
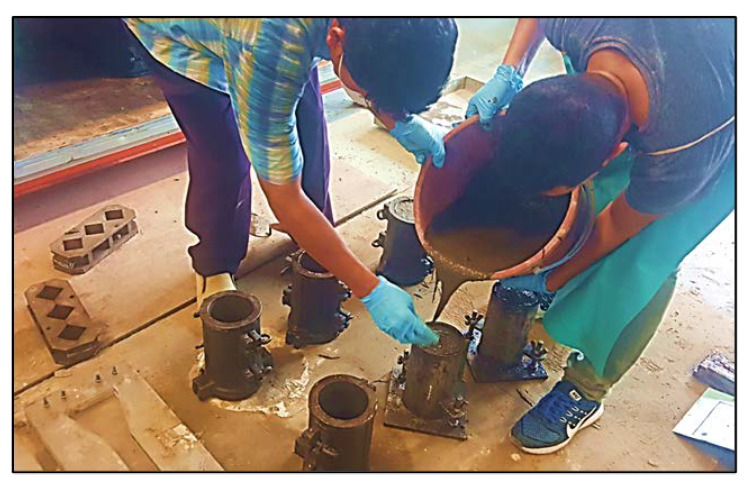
ECC casting on the cylinder molds.

Initially, the solid ingredients comprising of cement, fly ash and fine sand were mixed in a dry state for about 1 min only. Water and superplasticizer were then added to the dry mixture and further mixed for another 4–5 min in a rotating mixer until the blend became consistent and homogeneous. Afterward, silica fume and quartz powder are put together into the mixture and mixed to the required workability. Immediately, the related fibers were gradually and randomly introduced to the mortar mixture while the mixer was still rotating until all the fibers in the ECC mixture were fairly distributed. This phase spanned for another 2–3 min till the fibers were adequately distributed in the ECC mixture. The entire mixing process usually took for about 9 to 12 min. The fresh ECC mixture was cast in 100 mm diameter and 200 mm height of cylinder molds as shown in Figure 1. After 24 h, the specimens were dislodged from the molds and stored in a curing container in the lab at ambient temperature of (23 ± 2) °C.

### 2.4. Testing Procedure

**Figure 2:**
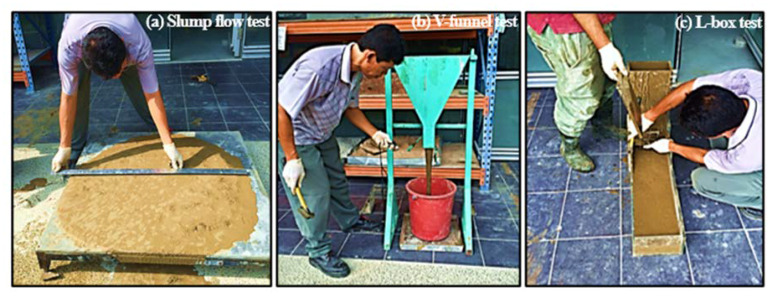
Views of the fresh properties tests: (**a**) slump flow test, (**b**) V-funnel test, (**c**) L-box test.

**Figure 3:**
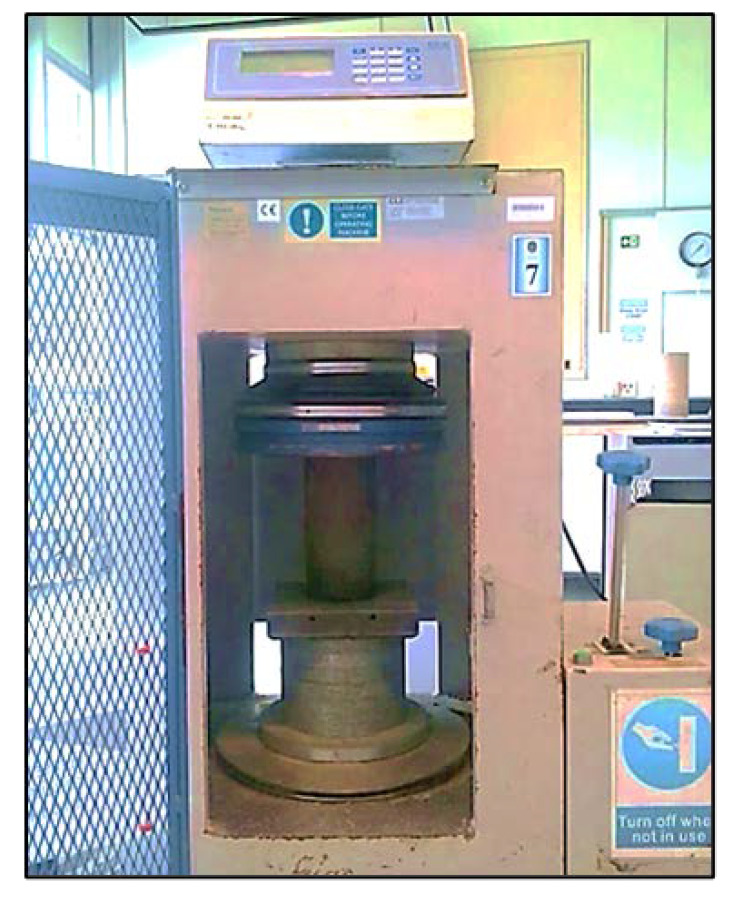
The experimental setup for the ECC compressive test.

Many codes have arranged the tests of the fresh properties of concrete, such as ASTM, BS EN, etc. However, up to present, no unique standard codes have arranged how to measure the workability of ECC. In this study, the fresh properties tests of ECC refer to the EFNARC [24] and European self-compacting concrete (SCC) guidelines [25]. Related to the performance of ECC physically, the ECC properties in the fresh state were examined based on the three different tests, which are the slump flow test, V-funnel test and L-box test. The fresh properties tests of ECC can be seen in Figure 2. Aside from that, a digital universal testing machine with a load capacity of 3000 kN (produced by ELE Group: Paraparaumu, New Zealand) shown in Figure 3 was used to measure the compressive strength of hardened ECC samples. Each cylinder sample test was exposed to a force at a loading rate of 2.4 kN/s until it failed. The compressive strength of specimens were evaluated at 1, 7, 14 and 28 days in accordance with ASTM C39 [26]. At each testing age, three specimens of each ECC type were used to conduct the compressive strength test.

## 3. Results and Discussion

### 3.1. Fresh Properties of ECC Mixes

In this study, the slump-flow and T500 time is a test to assess the flow ability and the flow rate of self-compacting ECC in the absence of obstructions. The test equipment required are the Abrams cone and the square steel table as illustrated in Figure 2a. The V-funnel test is a test that intended to assess the viscosity and filling ability of ECC. The V-funnel apparatus was constructed from a steel container with a flat horizontal top and watertight opening gate which could be momentarily released as shown in Figure 2b. Whereas, the L-box test is a test that intended to assess the passing ability of ECC to flow through the tight openings including spaces between steel bars and other obstructions without segregation or blocking. The L-box apparatus can be seen in Figure 2c.

Workability is highly demanded in the fresh concrete. Notwithstanding, the ECC is grouped as self-compacting concrete (SCC), so the viscosity and deformability of the fresh ECC can be examined through the measurement of its workability. This covers the slump flow test, filling ability of the V-funnel test, and passing ability of the L-box test. Commonly, the fresh property test results of all ECC mixes in this study exhibited that the slump flow diameter was ranged from 840–853 mm, T_500_ of 4–5 seconds, V-funnel time of 7–9 seconds, and L-box ratio of 0.92–0.97. All these measurements are in accordance with those specified by the related codes [24,25]. Apparently, the FR-ECC mixes showed the good workability and consistency.

The use of cement, quartz powder and silica fume can influence the workability of ECC mixes. These materials can absorb the water. However, the water absorption of cement and quartz powder are actually still much lower than silica fume. As reported by Holland [27], that ordinary Portland cement (OPC) having the surface area fineness of 370 m^2^/kg and Wang et al. [28] have stated that the Blaine fineness of quartz powder was 521 m^2^/kg. Admittedly, silica fume has the specific surface area of 15,000–30,000 m^2^/kg [27]. Aside from that, the use of water in all ECC mixes is constant. Basically, all these conditions can cause the low workability of the ECC mixes. However, by adjusting the superplasticizer (SP) content for each mix, the good consistency and workability of ECC mixtures can be achieved.

With respect to Table 5 to Table 8, the water-binder ratio is kept constant. It can be exhibited that the increment of the PVA volume fraction was always accompanied by increasing the SP content. Significantly, we obtained the good consistency and workability of the ECC mixes. This is in accordance with the use of fly ash in the mixes that improved the workability attributed by the spherical shape of fly ash particles with specific surface area of 420 m^2^/kg [27]. Similarly, due to the ball bearing effect, the fly ash particles can create the lubricating action and generate the composites with the high workability [29,30,31]. The PVA fibers have hydrophilic behavior [32,33,34,35]. These fibers can absorb the water. Hence, the PVA fibers can decrease the workability of ECC mixes.

### 3.2. Compressive Strength of PVA Fibers-Based ECC

**Figure 4:**
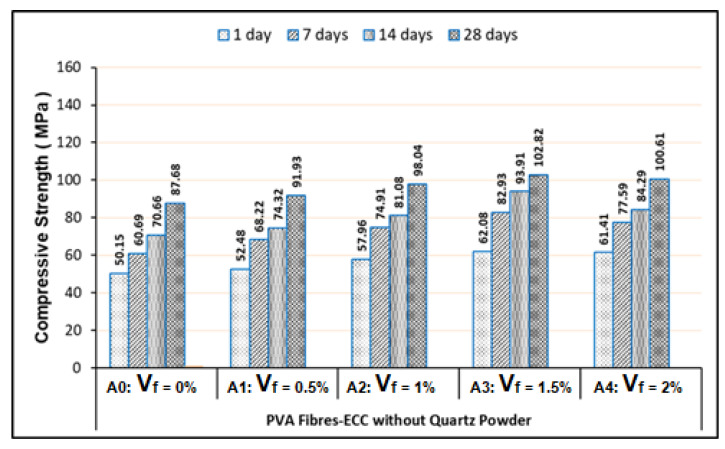
Compressive strength of the PVA fibers-ECC without quartz powder.

By comparing with ECC-2 investigated by Mohammed et al. [23], for the Mixes-A (A0–A4) in this study, the compressive strength of ECC samples exhibited decrements of 2%–3%. Even though the water-binder ratio has become lower from 0.175 to 0.155, decrements of the silica fume content of 27.65% can become its cause. As known, silica fume (SF) is very rich in SiO_2_, about 97.2%, which causes SF to become a very reactive pozzolanic material in the composites. Therefore, decrement of the silica fume amount in this study can lead to decrement of the ECC strength compared to the test results of Mohammed et al. [23]. Nevertheless, increment of the fly ash content of 14.69% also can be a cause of decreasing the compressive strength comparing to the test results of Mohammed et al. [23]. Such conditions can be explained by the statement of Haque and Kayali [36], that the strength of concrete/composites can be affected by the slow pozzolanic reaction of fly ash (FA). The compressive strength of Mixes-A (PVA Fibers-ECC without quartz powder) are exhibited in Figure 4.

Related to the use of PVA fibers, the compression test results have shown that the strength improvement of the ECC effectively occurred in the mixtures using the PVA fiber contents with the V_f_ of 0.5%, 1% and 1.5%. The optimum compressive strength had been reached by the mixtures with the V_f_ of 1.5%. Whereas for the mixtures with the V_f_ larger than 1.5%, which was 2% of their compressive strengths that were decreased, as illustrated in Figure 4. Therefore, the PVA fiber contents with the V_f_ of 2% in the ECC mixtures were excessive, which indicates that it had occurred as a result of the water being consumed by the PVA fibers during the cement hydration or the pozzolanic reaction process, so it may have effect the CSH formation. Consequently, the excessive amount of PVA fibers in the ECC mixtures were not effective related to the fiber dispersion and orientation in the ECC matrix, where this situation could produce an insufficient bonding strength between the PVA fibers and matrix. Hence, the compressive strength of ECC samples decreased when volume fraction of PVA fibers larger than 1.5%. The same thing also happened on the PVA-ECC samples using quartz powder, in which the optimum compressive strength was achieved at V_f_ of 1.5%.

**Figure 5:**
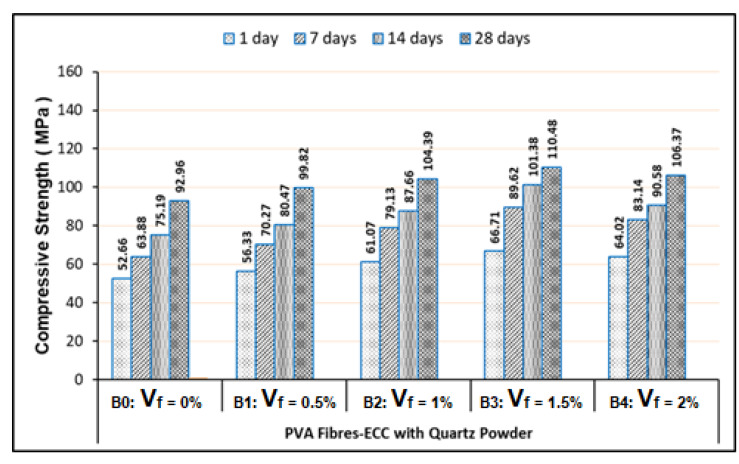
Compressive strength of the PVA fibers-ECC with quartz powder.

Based on the test results, as shown in Figure 5, the addition of quartz powder into the PVA fibers-ECC mixtures can improve the composites strength. In this part, the quartz powder addition can increase the average compressive strength of 6.85% or the average strength ratio of 1.0685 for the ECC curing age of 28 days, compared to the ECC samples without quartz powder (as exhibited in Figure 4). Referring to the chemical content aspect, basically the quartz powder characteristic (with SiO_2_ content of 96.2%) is similar to the silica fume. Potentially therefore, the quartz powder addition also can increase the ECC strength. In addition, based on the particle size aspect, the quartz powder is smaller and finer than cement and fly ash. Holland [27] has reported that the average particle size of cement is about 45 μm and less than 0.1 μm for silica fume. Then, the typical particle size of fly ash is around 20 μm [37]. Meanwhile, the average particle size of quartz powder was 4.56 µm [38]. Thus, the quartz powder particles can fill cavities contained in the fly ash and cement particles, which makes the composite to be denser and more compact. By comparing the test results on the PVA-ECC samples as shown in Figure 5 and Figure 4, it can be stated that the micro-filler effect of the quartz powder particles can contribute higher strength on the ECC composites.

### 3.3. Compressive Strength of Steel Fibers-Based ECC

**Figure 6:**
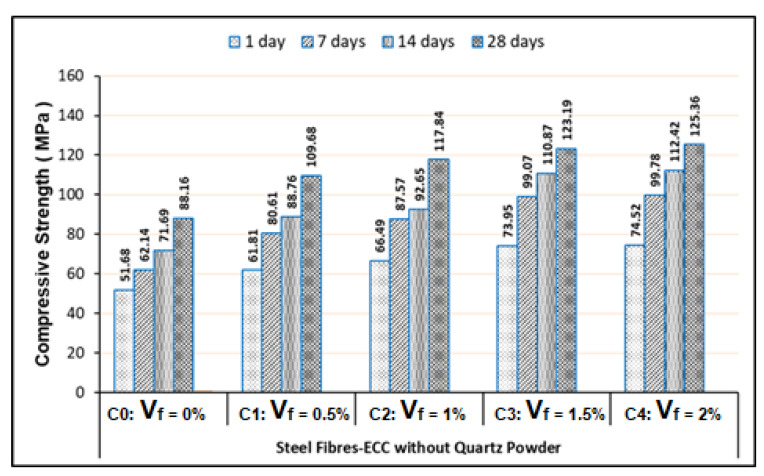
Compressive strength of the Steel fibers-ECC without quartz powder.

**Figure 7:**
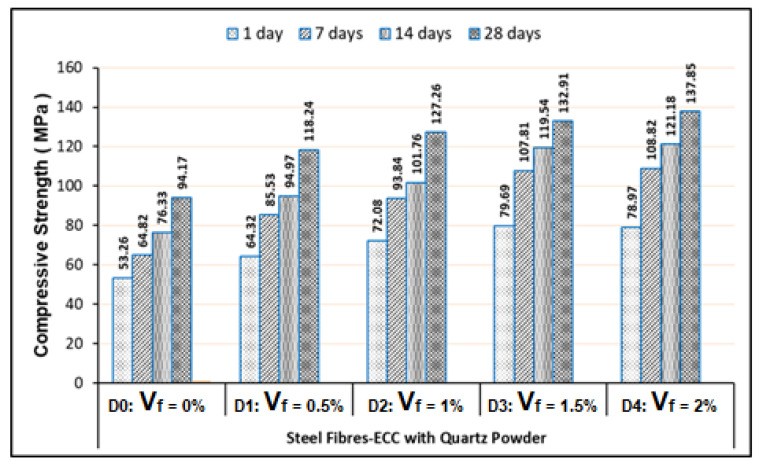
Compressive strength of the steel fibers-ECC with quartz powder.

The micro-steel fibers used in this study were unique. They have very high tensile strength of 2300 MPa and the steel fiber type is with the hooked ends. This condition can cause the Mixes-C (C0–C4) have higher strength compared to the Mixes-A (A0–A4), as shown in Figure 4 and Figure 6. Additionally, the Mixes-D (D0–D4) have greater strength than the Mixes-B (B0–B4), as exhibited in Figure 5 and Figure 7. The hooked end of steel fibers can provide an adequate anchorage strength. Unlike PVA fibers, the steel fibers are not categorized as hydrophilic. Hence, the SP contents of the steel fibers-based ECC mixes are lower than the PVA fibers-based ECC mixes, as presented in Table 5 to Table 8. Pertaining to the use of the steel fibers in ECC, workability and consistency of all ECC mixes is quite supportive, so it will not provide the balling effect, even though the V_f_ of steel fibers reaches 2%. All these conditions cause the compressive strength to achieve for the steel fibers-based ECC mixes with V_f_ of 2% having the highest strength compared to other volume fractions, as shown in Figure 6 and Figure 7.

Based on the compression test results, as exhibited in Figure 7, the addition of quartz powder into the steel fibers-based ECC mixtures can improve the ECC strength. In this section, the quartz powder addition can enhance the average compressive strength of 8.09% or the average strength ratio of 1.0809 for the ECC curing age of 28 days, compared to the ECC samples without quartz powder (as exhibited in Figure 6). Finally, due to addition of quartz powder on the PVA and steel fibers ECC samples, we found higher compressive strength with the average strength ratio of 1.0747, compared to the FR-ECC samples without quartz powder. The increased compressive strength is due to the micro-filler effect of quartz powder on the ECC composites. The explanation for this is the same as what has been described for the PVA fibers-based ECC using quartz powder, aforementioned. Furthermore, the steel fibers-ECC samples have higher compressive strength than PVA fibers-ECC samples, with the average strength ratio of 1.1760. This is attributed to the steel fibers having higher tensile strength than PVA fibers, and due to the anchorage strength contributed by the hooked-ends of steel fibers.

Zia et al. [39] reported that high early strength concrete means that the compressive strength of concrete at the first 24 h after casting could achieve structural concrete quality (compressive strength) of at least 35 MPa, whereas Li and Li [40] described that the high early concrete strength is minimum 42.30 MPa, and then Holland [27] stipulated that at least 27.56 MPa to achieve the high early strength of concrete.

Referring to our test results presented in Figure 4 to Figure 7, they were exhibited that the minimum compressive strengths of all FR-ECC samples for 1-day curing age are at least 50.15 MPa. Therefore, all FR-ECC samples in this study can be categorized as the high-early strength composites. In addition to the sample testing on 1-day curing age, in this study, the compressive strength of hardened FR-ECC samples was also measured at the testing ages of 7, 14 and 28 days. The compressive strength had experienced drastic changes within 28 days as presented in Figure 4 to Figure 7. This was attributed to the cement hydration process which took place fast to form the calcium silicate hydrate (CSH). However, the increment of the concrete strength can continue to occur after 28 days, but usually this process will take place very slowly.

### 3.4. The Failure Condition of ECC Samples

**Figure 8:**
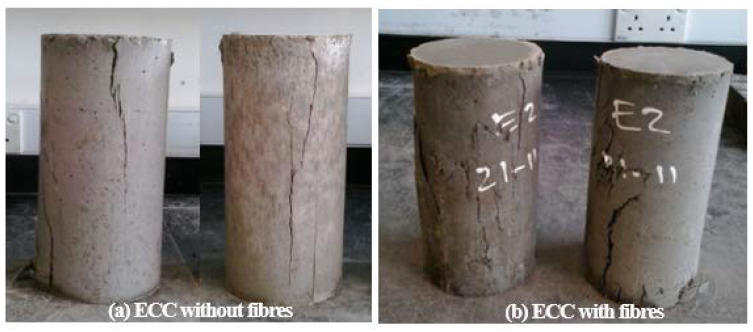
Typical failure condition of ECC samples: (**a**) for ECC without fibres, (**b**) for ECC with fibres.

The typical failure conditions of ECC samples after the compression test are shown in Figure 8. Unlike the ECC samples without fibers, the failures of ECC samples with fibers were always preceded by the bulging process. The smeared cracks appear during the compression loading up to fail. Due to the pozzolanic reaction, averaging the fibers dispersion and orientation in the ECC matrices, the fibers-based ECC samples were able to contribute more strength at their cementitious matrices, and also no spalling occurred at the time of failure.

## 4. Conclusions

Based on results of above studies, the following conclusions can be drawn:

1. Unlike the steel fibers, PVA fibers have hydrophilic behavior. PVA fibers can affect the workability and consistency of ECC mixes. For the use of constant water-binder ratio, the increment of the PVA volume fraction can cause enhancement of the SP content.
2. The fresh mixes of the PVA and steel fibers-based ECC have good consistency and workability. The FR-ECC mixes can be categorized as the self-compacting composites, as stipulated by the used standard codes.
3. The minimum compressive strengths of all FR-ECC samples for 1-day curing age are at least 50.15 MPa. Based on the used references, all FR-ECC samples in this study can be categorized as the high-early strength composites.
4. For the PVA fibers-based ECC, the quartz powder addition can increase the average compressive strength of 6.85% or the average strength ratio of 1.0685 for the ECC curing age of 28 days compared to the PVA fibers-based samples without quartz powder. On the other hand, for the steel fibers-based ECC, the addition of quartz powder can enhance the average compressive strength of 8.09% or the average strength ratio of 1.0809 compared to the steel fibers-based ECC samples without quartz powder. Finally, due to the addition of quartz powder on the PVA and steel fibers ECC samples, higher compressive strength was generated with the average strength ratio of 1.0747, compared to the FR-ECC samples without quartz powder.
5. The steel fibers-ECC samples have higher compressive strength than PVA fibers-ECC samples, with the average strength ratio of 1.1760. This is attributed to the steel fibers having higher tensile strength than PVA fibers, and also due to the anchorage strength contributed by the hooked-ends of steel fibers.
6. Optimum compressive strength of the PVA fibers-based ECC was achieved at the volume fraction of 1.5%. Larger amounts of PVA fibers in the ECC mixtures were not effective related to the fiber dispersion and orientation in the PVA-ECC matrix, where this situation could produce an insufficient bonding strength between the PVA fibers and matrix.
7. Even though the volume fraction is 2%, the steel fibers-based ECC samples were able to produce the highest strength compared to other the steel fibers-based ECC samples with less volume fraction. Apparently, no balling effect occurs in the steel fibers-based ECC mixes.
8. The failures condition of the fiber-based ECC samples were always preceded by the bulging process and occurs the smeared cracks during the compression loading.
9. The coarse aggregates were not utilized in the FR-ECC owing to that they can cause an adverse effect on the unique ductile behavior of composites. FR-ECC is unique, more superior than the normal concrete and uses the low volume fraction of fibers (2% or less). Moreover, FR-ECC is suitable and can be applied for the seismic-resistant buildings, retrofit materials, and the disturbed regions such as beam-column joint, corbels, link-slab, deep beam, support region, dapped-end area, etc. Thus, it does not need a high cost of maintenance.

## Figures and Tables

**Table 1:** The main mechanical properties of engineered cementitious composites (ECC) [7].

Compressive Strength	First Crack Strength	Ultimate Tensile Strength	Ultimate Tensile Strain	Young’s Modulus	Flexural Strength	Density
(MPa)	(MPa)	(MPa)	(%)	(GPa)	(MPa)	(kg/m^3^)
20–95	3–7	4–12	1–8	18–34	10–30	950–2300

**Table 2:** Chemical composition of cementitious materials (percentage by weight).

Chemical Oxide	Cement (%)	Fly Ash (%)	Silica Fume (%)	Quartz Powder (%)
SiO_2_	11.80	35.80	97.20	96.20
Al_2_O_3_	1.88	14.00	0.00	0.29
Fe_2_O_3_	4.14	22.90	0.06	0.13
CaO	76.20	18.00	0.43	1.61
MgO	0.82	2.66	0.00	0.00
SO_3_	3.64	1.11	0.25	0.13
K_2_O	0.40	2.09	0.48	0.15
TiO_2_	0.20	1.45	0.00	0.00
MnO	0.12	0.25	0.00	0.00
P_2_O_5_	0.60	1.15	1.31	1.51

**Table 3:** Physical properties of polyvinyl alcohol (PVA) and steel fibers.

Fiber Type	Diameter	Fiber Length	Specific Gravity	Elastic Modulus	Tensile Strength
	(mm)	(MPa)		(GPa)	(MPa)
PVA: RFS400	0.2	18	1.3	29	1000
Steel: hooked-ends	0.2	20	7.86	200	2300

**Table 4:** Characteristic of superplasticizer (SP).

ID	Product Name	Appearance/Color	pH	Chemical Base
SP	VC-2044	Brownish liquid, clear to slightly cloudy	6.2 ± 0.5	Aqueous solution of modified polycarboxylates

**Table 5:** Mix proportion of PVA fibers-based ECC without quartz powder.

Mix No.	OPC (kg/m^3^)	FA (kg/m^3^)	SF (kg/m^3^)	Water (kg/m^3^)	WRS (kg/m^3^)	QP (kg/m^3^)	SP (kg/m^3^)	PVA (kg/m^3^)
A0	580.00	406.00	73.95	152.83	464.00	0.00	21.69	0.00
A1	580.00	406.00	73.95	152.83	464.00	0.00	22.19	6.50
A2	580.00	406.00	73.95	152.83	464.00	0.00	22.68	13.00
A3	580.00	406.00	73.95	152.83	464.00	0.00	23.17	19.50
A4	580.00	406.00	73.95	152.83	464.00	0.00	23.66	26.00

**Table 6:** Mix proportion of PVA fibers-based ECC with quartz powder.

Mix No.	OPC (kg/m^3^)	FA (kg/m^3^)	SF (kg/m^3^)	Water (kg/m^3^)	WRS (kg/m^3^)	QP (kg/m^3^)	SP (kg/m^3^)	PVA (kg/m^3^)
B0	580.00	406.00	73.95	152.83	464.00	24.65	22.68	0.00
B1	580.00	406.00	73.95	152.83	464.00	24.65	23.17	6.50
B2	580.00	406.00	73.95	152.83	464.00	24.65	23.66	13.00
B3	580.00	406.00	73.95	152.83	464.00	24.65	24.16	19.50
B4	580.00	406.00	73.95	152.83	464.00	24.65	24.65	26.00

**Table 7:** Mix proportion of steel fiber-based ECC without quartz powder.

Mix No.	OPC (kg/m^3^)	FA (kg/m^3^)	SF (kg/m^3^)	Water (kg/m^3^)	WRS (kg/m^3^)	QP (kg/m^3^)	SP (kg/m^3^)	Steel Fiber (kg/m^3^)
C0	580.00	406.00	73.95	152.83	464.00	0.00	20.71	0.00
C1	580.00	406.00	73.95	152.83	464.00	0.00	20.71	39.30
C2	580.00	406.00	73.95	152.83	464.00	0.00	20.71	78.60
C3	580.00	406.00	73.95	152.83	464.00	0.00	20.71	117.90
C4	580.00	406.00	73.95	152.83	464.00	0.00	20.71	157.20

**Table 8:** Mix proportion of steel fiber-based ECC with quartz powder.

Mix No.	OPC (kg/m^3^)	FA (kg/m^3^)	SF (kg/m^3^)	Water (kg/m^3^)	WRS (kg/m^3^)	QP (kg/m^3^)	SP (kg/m^3^)	Steel Fiber (kg/m^3^)
D0	580.00	406.00	73.95	152.83	464.00	24.65	21.69	0.00
D1	580.00	406.00	73.95	152.83	464.00	24.65	21.69	39.30
D2	580.00	406.00	73.95	152.83	464.00	24.65	21.69	78.60
D3	580.00	406.00	73.95	152.83	464.00	24.65	21.69	117.90
D4	580.00	406.00	73.95	152.83	464.00	24.65	21.69	157.20
